# Non-Small Cell Lung Cancer: Challenge and Improvement of Immune Drug Resistance

**DOI:** 10.3389/fonc.2021.739191

**Published:** 2021-08-31

**Authors:** Fanming Kong, Ziwei Wang, Dongying Liao, Jinhui Zuo, Hongxia Xie, Xiaojiang Li, Yingjie Jia

**Affiliations:** ^1^Department of Oncology, First Teaching Hospital of Tianjin University of Traditional Chinese Medicine, Tianjin, China; ^2^National Clinical Research Center for Chinese Medicine Acupuncture and Moxibustion, Tianjin, China

**Keywords:** non-small cell lung cancer, immunotherapy, drug resistance, strategy, biomarkers

## Abstract

Lung cancer is the leading cause of cancer deaths in the world. At present, immunotherapy has made a great breakthrough in lung cancer treatment. A variety of immune checkpoint inhibitors have been applied into clinical practice, including antibodies targeting the programmed cell death-1, programmed cell death-ligand 1, and cytotoxic T-lymphocyte antigen 4. However, in the actual clinical process, about 30%–50% of patients still do not receive long-term benefits. Abnormal antigen presentation, functional gene mutation, tumor microenvironment, and other factors can lead to primary or secondary resistance. In this paper, we reviewed the immune mechanism of immune checkpoint inhibitor resistance, various combination strategies, and prediction of biomarkers to overcome resistance in order to accurately screen out the advantageous population, expand the beneficiary population, and enable precise and individualized medicine.

## Background

This article provides an update on the global cancer burden using the GLOBOCAN 2020 estimates of cancer incidence and mortality produced by the International Agency for Research on Cancer. Worldwide, an estimated 19.3 million new cancer cases and almost 10.0 million cancer deaths occurred in 2020. Lung cancer is one of the most commonly diagnosed cancers after breast cancer, with an estimated 2.3 million (11.7%) new cases and 1.8 million (18%) deaths in 2020. Lung cancer has not only become the leading cause of cancer death. It is also the leading burden on global health care (1). Through the traditional treatment methods [radiotherapy (RT), chemotherapy, targeting], lung cancer patients have little benefit. Recently, immune checkpoint inhibitors (ICIs) have become the most promising treatment for several kinds of cancer, especially in lung cancer. Nivolumab, pembrolizumab, and atezolizumab have been approved by the Food and Drug Administration (FDA). However, as clinical use becomes more widespread, approximately 30%–50% of patients receiving first-line ICIs experience temporary or no benefit. Immune drugs can also be divided into endogenous and exogenous drug resistance. Endogenous drug resistance refers to drug resistance caused by changes in tumor cells themselves, such as abnormal antigen presentation, functional gene mutation and inactivation, reduced immunogenicity, and tumor microenvironment. Exogenous drug resistance refers to external factors that affect all processes of T-cell activation. Therefore, in the current era of precision medicine, it is an urgent problem to clarify the mechanism of drug resistance and screen the beneficiaries. In this review, the known mechanisms of immune resistance and potential therapeutic strategies to reverse immune resistance and to predict poor prognosis are reviewed.

## Drug Resistance Mechanism

### Abnormal Antigen Presentation

The activation of T cells requires two signals. The first signal is the T-cell receptor (TCR) signal formed by the combination of TCR and peptide-major histocompatibility complex (MHC) molecules, but this signal is not enough to activate resting T cells. Only in the case of the second costimulatory signal provided by CD28 and its receptor, T-cell activation-related RNA and proteins will be synthesized, the key cytokine interleukin (IL)-2 will be secreted, and cells will enter from G0 phase to G1 phase. Therefore, it is the costimulatory signal and TCR signal that complete the activation of T cells. Studies have shown that B2M, as an important part of human leukocyte antigen (HLA)-I molecules, participates in the folding and transport of MHC-I molecules and plays an important role in the processing and presentation of tumor antigens. B2M mutation can lead to impaired expression of MHC-I molecules on the surface of antigen-presenting cells (APCs) and then lead to impaired antigen presentation, resulting in immunotherapy resistance (2). In addition, negative costimulatory molecules such as cytotoxic T-lymphocyte antigen 4 (CTLA-4) and programmed cell death-1 (PD-1) and their ligands CD80, CD86, PD-L1, and PD-L2 can prevent the body from producing second signals, resulting in downregulation or termination of T-cell activation (3).

### Immune Cells: Tumor-Associated Macrophages, Myeloid-Derived Suppressor Cells, Regulatory T Cells

A large number of studies have shown that immune cells play a key role in tumor progression and inflammation. First, tumor-associated macrophages (TAMs) showed significant plasticity toward environmental cues (4, 5). In the early stage of the tumor, TAMS mainly showed M1 phenotype, while in the late stage, TAMS mostly belonged to M2 phenotype (6). M1 macrophages are pro-inflammatory cells, but they have an antitumor effect, which is related to the cytotoxicity and immunostimulatory function to cancer cells. M2 macrophages expressing anti-inflammatory cytokines such as IL-10, C-C motif chemokine ligand 22 (CCL22), and CCL18 can reduce inflammatory response but can promote tumors due to immunosuppression and angiogenesis induction (4, 7, 8). In tumors, microenvironment, such as hypoxia, nitric oxide (NO), can promote TAMs to M2 polarization. In addition, macrophage colony-stimulating factor (M-CSF) produced by tumor cells can also promote the polarization of TAMs to M2, resulting in tumor escape. The main secretion of M2 suppresses cytokines IL-10 and transforming growth factor-β (TGF-β), and the presence of antigen is weak, which inhibits T-cell activation and contributes to tumor immunity (7, 8).

Second, regulatory T (Treg) cells exert their immunosuppressive function through a variety of mechanisms. The high expression of IL-2 receptor on the surface of Treg cells can neutralize IL-2 to limit the proliferation and activation of T cells and produce inhibitory cytokines (TGF-β, IL-10, and IL-35) and cytotoxic substances (perforin and granulase) to inhibit and kill the excitation of effector T cells (9). CTLA-4 expressed by Treg cells binds to CD80/86 to impair APC maturation and inhibit T-cell proliferation, such as dendritic cells (DCs) (10). In addition, Treg cells had low expression of Nrf2, which is a key transcription factor of antioxidant reaction. Oxidative stress can induce apoptosis of Treg cells and release a large number of ATP, which is metabolized into adenosine by CD39 and CD73, which are highly expressed in Treg cells. Adenosine binds to A2A receptor (A2AR) to inhibit effector T cells (9, 11).

Last, myeloid-derived suppressor cells (MDSCs) can promote tumor growth through immunological inhibition and non-immunological inhibition, in which immunosuppressive mediator ARG1 and inducible NO synthase (INOS) can decompose L-arginine into 1-ornithine and urea, NO and nitrite, an important mediator of the IL-2 pathway, resulting in T-cell expression incompetence (12, 13). MDSC also expressed a high level of indoleamine 2mine3-dioxygenase (IDO), which can degrade 1-tryptophan to N-formylcanine, inhibit the proliferation and activation of T cells and NK cells, and promote CD4+ T cells to differentiate into Treg (14, 15). In addition, MDSC secretes immunosuppressive cytokines and growth factors (TGF-β and IL-10) to reduce the antitumor activity of effector T cells, recruit Treg cells, and increase reactive oxygen species (ROS) and NO in the microenvironment to inhibit the antitumor activity of natural killer (NK) cells and effector T cells (7, 16–18). The latest research shows that MDSCs can exert an immunosuppressive effect by upregulating PD-L1 (19). In addition, MDSCs can also promote tumor progression through non-immunological mechanisms (20). MDSCs can produce a large number of matrix metalloproteinases (MMPs), especially MMP9, to promote the infiltration of metastatic cells (21) and secrete high levels of vascular endothelial growth factor (VEGF) and basic fibroblast growth factor (bFGF) to promote angiogenesis (22). To sum up, MDSCs play an important role in the occurrence and development of tumors.

### Tumor Endothelial Cells

Solid tumors tend to secrete a variety of pro-angiogenic factors, such as VEGF, hepatocyte growth factor, and platelet-derived growth factor. In 1971, Folkman (23) proposed that tumor growth was angiogenesis dependent, with further research. From 1983 to 1989, Senger et al. (24) proposed vascular permeability factor (VPF)/VEGF and Ferrara et al. (25) established the important position of VEGF until Terman isolated and purified VEGFR2 in the 1990s. These results fully indicate that VEGF (VEGFR2) plays an important role in the process of tumor growth, recurrence, and metastasis. VEGF/VEGFR is expressed in most tumors, including non-small cell lung cancer (NSCLC), and it has been found to increase the risk of tumor recurrence, metastasis, and death. Angiogenic factors are continuously secreted in the tumor microenvironment, resulting in abnormal angiogenesis. On the one hand, neovascularization usually lacks some adhesion molecules, and the downregulation of adhesion molecules leads to T-cell extravasation (26). At the same time, circulating VEGF hinders the maturation and function of DCs and helps tumors escape immune surveillance. On the other hand, neovascularization cannot offset the increase in oxygen consumption, so the hypoxia environment will directly damage the function of tumor-infiltrating lymphocyte (TIL). In addition, on the one hand, hypoxia can upregulate the inhibitory signals of antitumor immune response, such as PD-L1, IDO, IL-6, and IL-10 (27).

On the other hand, hypoxia induces upregulation of chemokine expression, which makes Treg cells reenter the tumor (28, 29). In addition, hypoxia can also promote the polarization of TAM to M2-like phenotype of TAMs (30). To sum up, angiogenesis can participate in tumor growth and immune escape through a variety of ways.

### Functional Gene Mutation and Inactivation

PTEN gene plays an important role in maintaining cell proliferation, differentiation, and apoptosis. PTEN can inhibit phosphoinositide 3-kinase (PI3K) pathway, which plays a regulatory role in some key cell processes such as tumor survival and proliferation. On the other hand, the lack of PTEN expression can activate the PI3K–AKT pathway, thus reducing the infiltration of lymphoid T cells and reduce the tumor killing effect of effector T cells (31). Similar to Janus kinase (JAK), it plays an important role in cytokine signal transduction. The JAK protein tyrosine kinase family consists of four members: tyrosine kinase (TYK)2, JAK1, JAK2, and JAK3. Patients with JAK1/2 gene mutations may be resistant to primary immunotherapy (32). Although JAK2 mutant tumor cells can produce interferon (IFN)-γ, the JAK2–signal transducer and activator of transcription _(STAT) signal pathway cannot be activated by IFN-γ and cannot upregulate the expression of PD-L1, which leads to the weak killing effect of IFN on JAK2 mutant tumor cells. However, the mutant cells of JAK1 were not sensitive to all kinds of effects of IFN. The above results suggest that the mutant tumor cells of the JAK1/JAK2 gene are not sensitive to the killing effect of IFN, and the expression level of PD-L1 is low, which makes the mutated tumor cells resistant to ICIs (33).

## Raising Drug Resistance

### Combined Application With Chemotherapy

Immune combined chemotherapy can not only increase the cross-presentation of antigens by dendritic cells (34) but also weaken the immunosuppressive components of the tumor microenvironment (35), such as Treg cells, MDSCs, immunosuppressive cytokines, etc., and then increase toxic lymphocytes and the ratio of Treg cells (36). In 2019, the American Society of Clinical Oncology (ASCO) published a three-phase clinical trial KEYNOTE-189 to evaluate the efficacy of NSCLC first-line treatment for advanced non-squamous NSCLC: pablizumab combined with chemotherapy compared with chemotherapy alone. The results showed that the immune combined chemotherapy group could significantly double the levels of overall survival time (OS), progression-free survival (PFS), and PFS2 [mean OS (mOS): 22.0 *vs*. 10.7 months, hazard ratio (HR): 0.56, 95% confidence interval (CI): 0.45–0.70; mean PFS (mPFS): 9.0 *vs*. 4.9 months, HR: 0.48, 95% CI: 0.40–0.58; and mPFS2: 17.0 *vs*. 9.0 months, HR: 0.49, 95% CI: 0.40–0.59]. From the safety analysis, the incidence of treatment-related select adverse events (AEs) of any grade in the combination chemotherapy group and the simple chemotherapy group was 26.4% *vs*. 12.9%. The grade 3–5 treatment-related select AEs were 10.9% *vs*. 4.5%. It further confirmed the safety and efficacy of this first-line chemotherapy regimen combined with pablizumab in the treatment of non-squamous NSCLC (37). In addition, KEYNOTE407 3-year follow-up data were released at this year’s European Lung Cancer Congress (ELCC) to evaluate the efficacy of immunotherapy combined with chemotherapy and chemotherapy alone. Studies have shown that immunotherapy combined with chemotherapy provides more lasting benefits to patients than chemotherapy (mOS: 17.1 *vs*. 11.6 months, HR: 0.71, 95% CI: 0.58–0.88; mPFS: 8.0 *vs*. 5.1 months, HR: 0.57, 95% CI: 0.47–0.69; mPFS2: 13.8 *vs*. 9.1 months, HR: 0.59, 95% CI: 0.49–0.72). Three-year follow-up data showed that OS: 29.7% *vs*. 18.2%, PFS: 16.1% *vs*. 6.5%; in terms of safety, the two groups of 3 and above treatment related adverse reactions were 74.8% *vs*. 70.0% (38). In the latest Camel-sq study released by the ELCC in 2021, carrizumab combined with carboplatin and paclitaxel combined with carboplatin chemotherapy regimen in the treatment of advanced non-small cell lung squamous cell carcinoma, objective response rate (ORR) and PFS were significantly prolonged (ORR: 64.8% *vs*. 36.7%, PFS: 8.5 *vs*. 4.9 months) (39).

### Combined Radiotherapy With Immunotherapy

Preclinical evidence points to RT as a priming event for immunotherapy. By modulating the host’s immune system, RT can render tumor cells more susceptible to T cell-mediated attack. RT promotes the release of tumor neoantigens from dying tumor cells, enhances MHC-I expression, and upregulates chemokines, cell adhesion molecules, and other immunomodulatory cell surface molecules, thereby potentiating an antitumor immune response by triggering immunogenic cell death (40). Just as the PACIFIC trial evaluated the efficacy of durvalumab consolidation therapy in patients with NSCLC after simultaneous RT and chemotherapy, the results showed that the mPFS was 16.9 *vs*. 5.6 months (HR: 0.55, 95% CI: 0.45–0.68), and the mOS was 47.5 *vs*. 28.1 months (HR: 0.55, 95% CI: 0.45–0.68) (41). In addition, a retrospective study at the 2020 ASCO conference showed that local treatment can significantly improve survival benefits (42). After immune resistance, local therapy combined with immunotherapy can reverse drug resistance to some extent, providing new treatment ideas for patients with immune drug resistance.

### Combined With Targeted Drugs

Anti-angiogenic therapy can change the function of tumor vascular endothelial cells to regulate immunosuppression and reduce the inhibitory effect of VEGF on DC migration and immune function (43, 44). Studies have shown that CTLA-4 or PD-1 inhibitors can reduce tumor vascular density, improve vascular perfusion, relieve hypoxia of tumor tissue, normalize blood vessels, and reduce the immunosuppressive effect of Treg, TAMS, and MDSCs (44–46). Therefore, the combination of immunosuppressive and anti-angiogenic drugs may play a synergistic role. As the latest results of the IMpower150 trial were presented at the American Association for Cancer Research (AACR) 2020 meeting of the AACR, the mOS of ABCP (atezolizumab+carboplatin+paclitaxel+bevacizumab) *vs*. BCP (carboplatin+paclitaxel+bevacizumab) was 19.5 *vs*. 14.7 months (HR: 0.80, 95% CI: 0.67-0.95), the mPFS was 8.4 *vs*. 6.8 months, and the ORR was 63.5% *vs*. 48.0%, mOS was 29.4% *vs*. 18.1%, respectively. In the liver metastasis subgroup, the mOS of ABCP *vs*. BCP was 13.2 *vs*. 9.1 months, and the PFS was 8.2 *vs*. 5.4 months. From the data survey, it can be seen that compared with BCP, ABCP can significantly improve the PFS and OS of patients (47, 48). From the above trial results, for epidermal growth factor receptor (EGFR) and anaplastic lymphoma kinase (ALK) mutant population, the combination of four drugs can bring significant survival benefits to patients with advanced NSCLC (49).

The Phase I/II KRYSTAL-1 test (NCT03785249) included patients with KRASG12C mutation-positive, unresectable, or metastatic NSCLC. After adagrasib monotherapy, the results showed that in patients with NSCLC, the overall remission rate of adagrasib treatment reached 45% and the disease control rate (DCR) reached 96%, and for NSCLC patients with STK11 mutation, the overall remission rate reached 64%. In terms of safety, the most common AEs of adagrasib treatment included nausea (54%), diarrhea (51%), vomiting (35%), and fatigue (32%) (50, 51). The researchers indicated that they plan to verify the efficacy of adagrasib in combination with other drugs or treatments in future trials, such as pembrolizumab, Keytruda (52). The Phase II KRYSTAL-7 trial (NCT04613596) of adagrasib combined with pimumab is under preparation, and the results are worth looking forward to. And now there are more and more data supporting the combination of immunotherapy and other targeted treatments.

## Combined Application of Double Immunity

PD-1 is highly expressed on T cells and interacts with its ligands PD-L1 and PD-L2 to inhibit T-cell activation and proliferation (53). CTLA-4 can reduce T-cell activity to maintain immune tolerance and homeostasis (54). The two interact to enhance the efficacy of immunotherapy. For example, in patients with advanced melanoma, the results showed that the ORR and PFS of double immunotherapy were higher than those of single-drug immunotherapy (55, 56). The Checkmate-227 test was used to evaluate the efficacy of dual immunotherapy and chemotherapy alone in the treatment of advanced NSCLC. The study showed that the OS and duration of response (DOR) of patients with PD-L1 ≥1% and PD-L1 <1% were significantly longer than those in the chemotherapy group (PD-L1-positive patients: mOS was 17.1 *vs*. 14.9 months, ORR value was 31.9% *vs*. 30%; mOS was 17.2 *vs*. 12.2 months, ORR value was 27.3% *vs*. 23.1%) (57, 58). In addition, like the Checkmate-227 study, the Checkmate-9LA trial, presented at the 2020 World Conference on Lung Cancer (WCLC), aims to evaluate the efficacy of chemotherapy alone or navulizumab combined with two cycles of chemotherapy in the treatment of metastatic NSCLC in Asian populations. The results showed that no matter what the expression of PD-L1 was, the combination of immunotherapy and chemotherapy could improve the expression of OS, without considering the expression of PD-L1. According to the expression analysis of PD-L1, the risk of death of patients with PD-L1 <1% (HR: 0.62, 95% CI: 0.45~0.85) and PD-L1 ≥1% (HR: 0.64, 95% CI: 0.5~0.82) decreased by 38% *vs*. 36% (HR: 0.64, 95% CI: 0.5~0.82) (59, 60). In addition, for the study of immune checkpoints, some new checkpoints have been explored, such as T-cell immunoreceptor with immunoglobulin (Ig) and ITIM domains (TIGIT), IDO, and lymphocyte activation gene (LAG)-3. Among the many new combinations and new targets, T-cell immune receptor (TIGIT) inhibitors carrying Ig and ITIM domains have attracted particular attention. A phase II study of atrizumab combined with TIGIT inhibitor tiragolumab *vs*. placebo combined with atrizumab was released at the 2020 ASCO Congress (61). The main characters were ORR and PFS. The results showed that for people with PDL1 >50%, tiragolumab+atrizumab significantly increased ORR and PFS time in the intention-to-treat (ITT) population compared with those in the control group, but for 1%~49% of PD-L1, the benefit of ORR and PFS time was limited. This study is still under further study, and the results are worth looking forward to.

## Discussion

Although the research ideas of immunotherapy drug resistance emerge endlessly, there are also some problems that cannot be ignored in the clinical trials of reversing immune drug resistance. In recent years, with the rapid development of medicine, we advocate “individualized medical treatment” and “precision treatment” in the field of oncology. Biomarkers have important clinical significance for the discovery, treatment, and prognosis of tumors. At present, PD-L1 is the most commonly used marker for predicting the efficacy of immunotherapy, but it still has some limitations and cannot be used as a routine marker in the clinic. Other related studies have shown that tumor mutation load, TILs, and microsatellite instability are important biomarkers to predict the efficacy of immunotherapy.

According to the retrospective analysis of CHECKMate-026 and the survival data of CHECKMate-227, the efficacy of immunotherapy in patients with high tumor mutation burden (TMB) was significantly better than that of chemotherapy (57, 62). In 2019, the National Comprehensive Cancer Network (NCCN) guidelines recommended TMB as one of the indicators to evaluate the efficacy of immunotherapy for NSCLC. At the AACR 2019 meeting, Wang et al. (63) reported the latest research results on the blood-based tumor mutant (bTMB) and realized bTMB’s effective prediction of PFS, especially OS, by redefining bTMB. However, due to the influence of different critical values, different sampling time, tumor heterogeneity, and other factors, TMB may still be a biomarker for clinical reference, and the selection of its effective threshold and the feasibility of detection need to be further verified by clinical data. Also at the ASCO conference in 2019, Johns Hopkins and Memorial Sloan Kettering Cancer Celter (MSKCC) published the results of trials on patients with NSCLC who received nivolumab before operation, suggesting that circulating tumor DNA (CtDNA) clearance and peripheral blood T-cell expansion can be used as biomarkers to predict recurrence and treatment efficacy (64). The WCLC in 2021 also reported on CtDNA. In addition, new biomarkers are being discovered. For example, a retrospective trial was conducted in Japan to evaluate the efficacy of nivumab or pembrolizumab in patients with advanced NSCLC with positive antibodies (rheumatoid factor, antinuclear antibodies, thyroid antibodies) before selection. The results showed that the ORR, DCR, and PFS of antibody-positive patients were significantly better than those of non-antibody-positive patients. Although the sample size included in this study is relatively small, its research is worthy of further study (65).

At present, with the further study of biomarkers, some studies also have found that Tumor Protein p53 (TP53) or Kirsten Rat Sarcoma Viral Oncogene Homolog (KRAS) gene mutations can increase the expression of PD-L1 and CD8+ T-cell infiltration; when both mutations are present, the expression of PD-L1 will be more significant, the tumor mutation load is often high, and the clinical benefits of pembrolizumab therapy are often better. Therefore, KRAS or TP53 mutations combined with PD-L1 or CD8+ T cells may be of better predictive value (66).

## Conclusion

There is still a certain problem of drug resistance in the clinical application of immunotherapy for NSCLC, although the combination of drugs can expand the effect of immunotherapy and improve immune drug resistance. However, based on these results, there are still several important problems to be solved: how to accurately grasp the dose and time sequence of the combination, and the choice of the dominant population is the key to improving the efficacy of drugs. The development of immunotherapy is a great breakthrough in tumor therapy and the cornerstone of advanced NSCLC therapy. In the future, looking for biomarkers to predict the efficacy of immunotherapy can make immunotherapy individualized and accurate. The research and development of new drugs will further improve the prognosis of patients with lung cancer (**Table 1**).

**Table 1:** Data on the results of trials related to non-small cell lung cancer.

Study (NCT-number)	Trial group	Trial design	Primary end points	Secondary end point	Adverse reactions greater than or equal to grade 3
Impower150 (46, 47), (NCT02366143)	Previously untreated non-squamous NSCLC patients without EGFR/ALK aberrations	Atezolizumab+Carboplatin+Paclitaxel+Bevacizumab (ABCP); Atezolizumab+Carboplatin+Paclitaxel (ACP); Carboplatin+Paclitaxel+ Bevacizumab (BCP)	PFS (Teff-high WT): NR*	ORR: NR*	Gr 3–4 treatment-related AEs occurred in 43%, 57%, and 49% of patients in ABCP, ACP, and BCP, respectively.
			OS (ITT-WT): median, ACP *vs*. BCP = 19.0 *vs*. 14.7 months median, ABCP *vs*. BCP = 19.5 *vs*. 14.7 months ABCP group is of great significance to liver metastasis and EGFR mutation	DOR: NR*	
Keynote189 (36), (NCT02578680)	Patients with advanced non-squamous (NSCLC) without an EGFR/ALK alteration	Pembrolizuab+Chemotherapy; Placebo+Chemotherapy	OS: median, 22.0 *vs*. 10.7 months	ORR: 48.3% *vs*. 19.9%	Gr 3–5 immune-mediated adverse reactions and infusion-related reactions occurred in 10.9% and 4.5%, respectively
			PFS: median, 9.0 *vs*. 4.9 months	DOR: median,12.4 *vs*. 7.1	
				PFS2: median,17.0 *vs*. 9.0 months	
Keynote407 (37), (NCT02775435)	Patients with previously untreated metastatic squamous NSCLC	Pembrolizuab+Chemotherapy; Placebo+Chemotherapy	OS: median, 17.2 *vs*. 11.6 months	ORR: 62.6% *vs*. 38.4%	Gr 3–5 treatment-related AEs occurred in 74.1% *vs*. 69.6%
			PFS: median, 8.0 *vs*. 5.1 months	DOR: 8.8 *vs*. 4.9months	
				PFS2: 13.8 *vs*. 9.1months	
PACIFIC (40), (NCT02125461)	Patients with unresectable stage III NSCLC without progression after chemoradiotherapy	Durvalumab+Chemotherapy; Placebo+Chemotherapy	OS: median, 47.5 *vs*. 28.1 months	NR*	NR*
			PFS: median, 16.9 *vs*. 5.6 months		
Checkmate-227 (56, 57), (NCT02477826)	Patients with unresectable stage III NSCLC without progression	Nivolumab+Ipilimumab; Chemotherapy	OS: (PD-L1 ≥1%): median, 17.1*vs*. 14.9 months (PD-L1 ≥50%): median, 21.2 *vs*. 14.0 months (PD-L1 <1%): median, 17.2 *vs*. 12.2 months	DOR: (PD-L1 ≥1%): 23.2 *vs*. 6.7 months; (PD-L1 ≥50%): 31.8 *vs*. 5.8 months; (PD-L1 <1%): 18.0 *vs*. 4.8 months	Gr 3–4 treatment-related AEs occurred in 33% *vs*. 36%
Checkmate-9LA (58, 59), (NCT03215706)	Patients with treatment-naive, histologically confirmed stage IV or recurrent NSCLC without an EGFR/ALK alteration	Nivolumab+Ipilimumab+Chemotherapy; Chemotherapy	OS: median, 15.8 *vs*. 11.0 months	ORR: 38% *vs*. 25.4%	Gr 3–5 treatment-related AEs occurred in 47% *vs*. 38% (The incidence of blood toxicity was higher in the chemotherapy group than in the chemotherapy group)
			PFS: median, 6.7 *vs*. 5.3 months	DOR: 13.0 *vs*. 5.6 months	
Checkmate-026 (61), (NCT02041533)	Patients with untreated stage IV or recurrent NSCLC and a PD-L1 tumor-expression level of 1% or more	Nivolumab; Chemotherapy	PFS (PD-L1 ≥5%): median, 4.2 *vs*. 5.9 months	OS: 14.4 *vs*. 13.2 months	Gr 3 treatment-related AEs occurred in 71% *vs*. 92%; Gr 4 treatment-related AEs occurred in 18% *vs*. 51%
Camel-sq (38), (NCT03668496)	Chinese patients with advanced non-squamous NSCLC and negative EGFR/ALK mutation	Camrelizumab+Chemotherapy; Placebo+Chemotherapy	OS: median, NR* *vs*. 14.5 months	ORR: 64.8% *vs*. 36.7%	Gr 3-5 treatment-related AEs occurred in 73.6% *vs*. 73.6%
			PFS: median, 8.5 *vs*. 4.9 months	DOR: median, 13.1 *vs*. 4.4 months	

*There is no report in the recently updated data of the trial.

## Author Contributions

FK and ZW contributed equally to this work. Other authors offer advice. All authors contributed to the article and approved the submitted version.

## Funding

This work is supported by the National Natural Science Foundation of China (No. 81403220 and No. 81904151), Tianjin Health and family planning-high level talent selection and training project, Tianjin Science and Technology Plan Projects (No. 17ZXMFSY00190), and Tianjin Traditional Chinese Medicine Research Project, Tianjin health and family planning commission (No. 2017003).

## Conflict of Interest

The authors declare that the research was conducted in the absence of any commercial or financial relationships that could be construed as a potential conflict of interest.

## Publisher’s Note

All claims expressed in this article are solely those of the authors and do not necessarily represent those of their affiliated organizations, or those of the publisher, the editors and the reviewers. Any product that may be evaluated in this article, or claim that may be made by its manufacturer, is not guaranteed or endorsed by the publisher.

## References

[1] Sung H Ferlay J Siegel RL Laversanne M Soerjomataram I Jemal A. Global Cancer Statistics 2020: GLOBOCAN Estimates of Incidence and Mortality Worldwide for 36 Cancers in 185 Countries. CA Cancer J Clin (2021) 71(3):209–49. 10.3322/caac.21660 33538338

[2] Nowicki TS Hu-Lieskovan S Ribas A. Mechanisms of Resistance to PD-1 and PD-L1 Blockade. Cancer J (2018) 24(1):47–53. 10.1097/PPO.0000000000000303 29360728 PMC5785093

[3] Xu W Hiếu T Malarkannan S Wang L. The Structure, Expression, and Multifaceted Role of Immune-Checkpoint Protein VISTA as a Critical Regulator of Anti-Tumor Immunity, Autoimmunity, and Inflammation. Cell Mol Immunol (2018) 15(5):438–46. 10.1038/cmi.2017.148 PMC6068175 29375120

[4] Delprat V Tellier C Demazy C Raes M Feron O Michiels C. Cycling Hypoxia Promotes a Pro-Inflammatory Phenotype in Macrophages *via* JNK/p65 Signaling Pathway. Sci Rep (2020) 10(1):882. 10.1038/s41598-020-57677-5 31964911 PMC6972721

[5] Palmieri EM Gonzalez-Cotto M Baseler WA Davies LC Ghesquière B Maio N. Nitric Oxide Orchestrates Metabolic Rewiring in M1 Macrophages by Targeting Aconitase 2 and Pyruvate Dehydrogenase. Nat Commun (2020) 11(1):698. 10.1038/s41467-020-14433-7 32019928 PMC7000728

[6] Singhal S Stadanlick J Annunziata MJ Rao AS Bhojnagarwala PS O'Brien S. Human Tumor-Associated Monocytes/Macrophages and Their Regulation of T Cell Responses in Early-Stage Lung Cancer. Sci Transl Med (2019) 11(479). 10.1126/scitranslmed.aat1500 PMC6800123 30760579

[7] Veglia F Perego M Gabrilovich D. Myeloid-Derived Suppressor Cells Coming of Age. Nat Immunol (2018) 19(2):108–19. 10.1038/s41590-017-0022-x PMC5854158 29348500

[8] Motallebnezhad M Jadidi-Niaragh F Qamsari ES Bagheri S Gharibi T Yousefi M. The Immunobiology of Myeloid-Derived Suppressor Cells in Cancer. Tumour Biol (2016) 37(2):1387–406. 10.1007/s13277-015-4477-9 26611648

[9] Ohue Y Nishikawa H Regulatory T. (Treg) Cells in Cancer: Can Treg Cells be a New Therapeutic Target? Cancer Sci (2019) 110(7):2080–9. 10.1111/cas.14069 PMC6609813 31102428

[10] Walker LS Sansom DM. The Emerging Role of CTLA4 as a Cell-Extrinsic Regulator of T Cell Responses. Nat Rev Immunol (2011) 11(12):852–63. 10.1038/nri3108 22116087

[11] Maj T Wang W Crespo J Zhang H Wang W Wei S. Oxidative Stress Controls Regulatory T Cell Apoptosis and Suppressor Activity and PD-L1-Blockade Resistance in Tumor. Nat Immunol (2017) 18(12):1332–41. 10.1038/ni.3868 PMC5770150 29083399

[12] Kowanetz M Wu X Lee J Tan M Hagenbeek T Qu X. ranulocyte-Colony Stimulating Factor Promotes Lung Metastasis Through Mobilization of Ly6G+Ly6C+ Granulocytes. Proc Natl Acad Sci USA (2010) 107(50):21248–55. 10.1073/pnas.1015855107 PMC3003076 21081700

[13] Yen BL Yen ML Hsu PJ Liu KJ Wang CJ Bai CH. Multipotent Human Mesenchymal Stromal Cells Mediate Expansion of Myeloid-Derived Suppressor Cells *via* Hepatocyte Growth Factor/C-Met and STAT3. Stem Cell Rep (2013) 1(2):139–51. 10.1016/j.stemcr.2013.06.006 PMC3757753 24052949

[14] Hu X Li B Li X Zhao X Wan L Lin G. Transmembrane TNF-α Promotes Suppressive Activities of Myeloid-Derived Suppressor Cells *via* TNFR2. J Immunol (2014) 192(3):1320–31. 10.4049/jimmunol.1203195 24379122

[15] Highfill SL Rodriguez PC Zhou Q Goetz CA Koehn BH Veenstra R. Bone Marrow Myeloid-Derived Suppressor Cells (MDSCs) Inhibit Graft-*Versus*-Host Disease (GVHD) *via* an Arginase-1-Dependent Mechanism That is Up-Regulated by Interleukin-13. Blood (2010) 116(25):5738–47. 10.1182/blood-2010-06-287839 PMC3031417 20807889

[16] Prima V Kaliberova LN Kaliberov S Curiel DT Kusmartsev S. COX2/mPGES1/PGE2 Pathway Regulates PD-L1 Expression in Tumor-Associated Macrophages and Myeloid-Derived Suppressor Cells. Proc Natl Acad Sci USA (2017) 114(5):1117–22. 10.1073/pnas.1612920114 PMC5293015 28096371

[17] Li L Wang L Li J Fan Z Yang L Zhang Z. Metformin-Induced Reduction of CD39 and CD73 Blocks Myeloid-Derived Suppressor Cell Activity in Patients With Ovarian Cancer. Cancer Res (2018) 78(7):1779–91. 10.1158/0008-5472.CAN-17-2460 PMC5882589 29374065

[18] Li J Wang L Chen X Li L Li Y Ping Y. CD39/CD73 Upregulation on Myeloid-Derived Suppressor Cells *via* TGF-β-mTOR-HIF-1 Signaling in Patients With non-Small Cell Lung Cancer. Oncoimmunology (2017) 6(6):e1320011. 10.1080/2162402X.2017.1320011 28680754 PMC5486179

[19] Berger KN Pu JJ. PD-1 Pathway and its Clinical Application: A 20year Journey After Discovery of the Complete Human PD-1 Gene. Gene (2018) 638:20–5. 10.1016/j.gene.2017.09.050 28951311

[20] Safarzadeh E Orangi M Mohammadi H Babaie F Baradaran B. Myeloid-Derived Suppressor Cells: Important Contributors to Tumor Progression and Metastasis. J Cell Physiol (2018) 233(4):3024–36. 10.1002/jcp.26075 28661031

[21] Jacob A Prekeris R. The Regulation of MMP Targeting to Invadopodia During Cancer Metastasis. Front Cell Dev Biol (2015) 3:4. 10.3389/fcell.2015.00004 25699257 PMC4313772

[22] Shen P Wang A He M Wang Q Zheng S. Increased Circulating Lin(-/Low) CD33(+) HLA-DR(-) Myeloid-Derived Suppressor Cells in Hepatocellular Carcinoma Patients. Hepatol Res (2014) 44(6):639–50. 10.1111/hepr.12167 23701406

[23] Folkman J. Endogenous Angiogenesis Inhibitors. Apmis (2004) 112(7-8):496–507. 10.1111/j.1600-0463.2004.apm11207-0809.x 15563312

[24] Senger DR Galli SJ Dvorak AM Perruzzi CA Harvey VS Dvorak HF. Tumor Cells Secrete a Vascular Permeability Factor that Promotes Accumulation of Ascites Fluid. Science (1983) 219(4587):983–5. 10.1126/science.6823562 6823562

[25] Ferrara N Henzel WJ. Pituitary Follicular Cells Secrete a Novel Heparin-Binding Growth Factor Specific for Vascular Endothelial Cells. Biochem Biophys Res Commun (1989) 161(2):851–8. 10.1016/0006-291x(89)92678-8 2735925

[26] Valach J Fík Z Strnad H Chovanec M Plzák J Cada Z. Smooth Muscle Actin-Expressing Stromal Fibroblasts in Head and Neck Squamous Cell Carcinoma: Increased Expression of Galectin-1 and Induction of Poor Prognosis Factors. Int J Cancer (2012) 131(11):2499–508. 10.1002/ijc.27550 22447203

[27] Reck M Garassino MC Imbimbo M Shepherd FA Socinski MA Shih JY. Antiangiogenic Therapy for Patients With Aggressive or Refractory Advanced Non-Small Cell Lung Cancer in the Second-Line Setting. Lung Cancer (2018) 120:62–9. 10.1016/j.lungcan.2018.03.025 29748017

[28] Curiel TJ Coukos G Zou L Alvarez X Cheng P Mottram P. Specific Recruitment of Regulatory T Cells in Ovarian Carcinoma Fosters Immune Privilege and Predicts Reduced Survival. Nat Med (2004) 10(9):942–9. 10.1038/nm1093 15322536

[29] Facciabene A Hagemann IS Balint K Barchetti A Wang L-P Gimotty PA. Tumour Hypoxia Promotes Tolerance and Angiogenesis *via* CCL28 and T(reg) Cells. Nature (2011) 475(7355):226–30. 10.1038/nature10169 21753853

[30] Movahedi K Laoui D Gysemans C Baeten M Stangé G Van den Bossche J. Different Tumor Microenvironments Contain Functionally Distinct Subsets of Macrophages Derived From Ly6C(high) Monocytes. Cancer Res (2010) 70(14):5728–39. 10.1158/0008-5472.CAN-09-4672 20570887

[31] Bucheit AD Chen G Siroy A Tetzlaff M Broaddus R Milton D. Complete Loss of PTEN Protein Expression Correlates With Shorter Time to Brain Metastasis and Survival in Stage IIIB/C Melanoma Patients With BRAFV600 Mutations. Clin Cancer Res (2014) 20(21):5527–36. 10.1158/1078-0432.CCR-14-1027 PMC4216767 25165098

[32] Shin DS Zaretsky JM Escuin-Ordinas H Garcia-Diaz A Hu-Lieskovan S Kalbasi A. Primary Resistance to PD-1 Blockade Mediated by JAK1/2 Mutations. Cancer Discovery (2017) 7(2):188–201. 10.1158/2159-8290.CD-16-1223 27903500 PMC5296316

[33] Zaretsky JM Garcia-Diaz A Shin DS Escuin-Ordinas H Hugo W Hu-Lieskovan S. Mutations Associated With Acquired Resistance to PD-1 Blockade in Melanoma. N Engl J Med (2016) 375(9):819–29. 10.1056/NEJMoa1604958 PMC5007206 27433843

[34] Bracci L Schiavoni G Sistigu A Belardelli F. Immune-Based Mechanisms of Cytotoxic Chemotherapy: Implications for the Design of Novel and Rationale-Based Combined Treatments Against Cancer. Cell Death Differ (2014) 21(1):15–25. 10.1038/cdd.2013.67 23787994 PMC3857622

[35] Wang Z Till B Gao Q. Chemotherapeutic Agent-Mediated Elimination of Myeloid-Derived Suppressor Cells. Oncoimmunology (2017) 6(7):e1331807. 10.1080/2162402X.2017.1331807 28811975 PMC5543863

[36] Roselli M Cereda V di Bari MG Formica V Spila A Jochems C. Effects of Conventional Therapeutic Interventions on the Number and Function of Regulatory T Cells. Oncoimmunology (2013) 2(10):e27025. 10.4161/onci.27025 24353914 PMC3862634

[37] Gadgeel S Rodríguez-Abreu D Speranza G Esteban E Felip E Dómine M. Updated Analysis From KEYNOTE-189: Pembrolizumab or Placebo Plus Pemetrexed and Platinum for Previously Untreated Metastatic Nonsquamous Non-Small-Cell Lung Cancer. J Clin Oncol (2020) 38(14):1505–17. 10.1200/jco.19.03136 32150489

[38] Paz-Ares L Luft A Vicente D Tafreshi A Gümüş M Mazières J. Pembrolizumab Plus Chemotherapy for Squamous Non-Small-Cell Lung Cancer. N Engl J Med (2018) 379(21):2040–51. 10.1056/NEJMoa1810865 30280635

[39] Zhou C Ren S Chen J Xu X Cheng Y Chen G. 96o Camrelizumab or Placebo Plus Carboplatin and Paclitaxel as First-Line Treatment for Advanced Squamous NSCLC (CameL-Sq): A Randomized, Double-Blind, Multicenter, Phase III Trial. J Thoracic Oncol (2021) 16(4S). 10.1016/S1556-0864(21)01938-9 34923163

[40] Ko EC Raben D Formenti SC. The Integration of Radiotherapy With Immunotherapy for the Treatment of Non-Small Cell Lung Cancer. Clin Cancer Res (2018) 24(23):5792–806. 10.1158/1078-0432.CCR-17-3620 29945993

[41] Faivre-Finn C Vicente D Kurata T Planchard D Paz-Ares L Vansteenkiste JF. Four-Year Survival With Durvalumab After Chemoradiotherapy in Stage III NSCLC—an Update From the PACIFIC Trial. J Thoracic Oncol (2021) 16(5):860–7. 10.1016/j.jtho.2020.12.015 33476803

[42] Ranjan P Atish M MVV Ravi S Erminia M. Acquired Resistance to PD-1/PD-L1 Blockade in Lung Cancer: Mechanisms and Patterns of Failure. Cancers (2020) 12(12). 10.3390/cancers12123851 PMC7767234 33419311

[43] Long J Hu Z Xue H Wang Y Chen J Tang F. Vascular Endothelial Growth Factor (VEGF) Impairs the Motility and Immune Function of Human Mature Dendritic Cells Through the VEGF Receptor 2-RhoA-Cofilin1 Pathway. Cancer Sci (2019) 110(8):2357–67. 10.1111/cas.14091 PMC6676124 31169331

[44] Tian L Goldstein A Wang H Ching Lo H Sun Kim I Welte T. Mutual Regulation of Tumour Vessel Normalization and Immunostimulatory Reprogramming. Nature (2017) 544(7649):250–4. 10.1038/nature21724 PMC5788037 28371798

[45] Huang Y Yuan J Righi E Kamoun WS Ancukiewicz M Nezivar J. Vascular Normalizing Doses of Antiangiogenic Treatment Reprogram the Immunosuppressive Tumor Microenvironment and Enhance Immunotherapy. Proc Natl Acad Sci USA (2012) 109(43):17561–6. 10.1073/pnas.1215397109 PMC3491458 23045683

[46] Gray JE Villegas A Daniel D Vicente D Murakami S Hui R. Three-Year Overall Survival With Durvalumab After Chemoradiotherapy in Stage III NSCLC-Update From PACIFIC. J Thorac Oncol (2020) 15(2):288–93. 10.1016/j.jtho.2019.10.002 PMC7244187 31622733

[47] Fukumura D Kloepper J Amoozgar Z Duda DG Jain RK. Enhancing Cancer Immunotherapy Using Antiangiogenics: Opportunities and Challenges. Nat Rev Clin Oncol (2018) 15(5):325–40. 10.1038/nrclinonc.2018.29 PMC5921900 29508855

[48] Reck M Mok TSK Nishio M Jotte RM Cappuzzo F Orlandi F. Atezolizumab Plus Bevacizumab and Chemotherapy in Non-Small-Cell Lung Cancer (IMpower150): Key Subgroup Analyses of Patients With EGFR Mutations or Baseline Liver Metastases in a Randomised, Open-Label Phase 3 Trial. Lancet Respir Med (2019) 7(5):387–401. 10.1016/S2213-2600(19)30084-0 30922878

[49] Socinski MA Jotte RM Cappuzzo F Orlandi F Stroyakovskiy D Nogami N. Atezolizumab for First-Line Treatment of Metastatic Nonsquamous NSCLC. N Engl J Med (2018) 378(24):2288–301. 10.1056/NEJMoa1716948 29863955

[50] Another KRAS Inhibitor Holds Its Own. Cancer Discov (2020) 10(12):Of2. 10.1158/2159-8290.CD-NB2020-098 33115812

[51] Jänne PA Rybkin II Spira AI Riely GJ Papadopoulos KP Sabari JK. KRYSTAL-1: Activity and Safety of Adagrasib (MRTX849) in Advanced/Metastatic Non–Small-Cell Lung Cancer (NSCLC) Harboring KRAS G12C Mutation. Eur J Cancer (2020) 138:S1–2. 10.1016/S0959-8049(20)31076-5

[52] Hong DS Fakih MG Strickler JH Desai J Durm GA Shapiro GI. KRAS(G12C) Inhibition With Sotorasib in Advanced Solid Tumors. N Engl J Med (2020) 383(13):1207–17. 10.1056/NEJMoa1917239 PMC7571518 32955176

[53] Weber JS D'Angelo SP Minor D Hodi FS Gutzmer R Neyns B. Nivolumab *Versus* Chemotherapy in Patients With Advanced Melanoma Who Progressed After Anti-CTLA-4 Treatment (CheckMate 037): A Randomised, Controlled, Open-Label, Phase 3 Trial. Lancet Oncol (2015) 16(4):375–84. 10.1016/S1470-2045(15)70076-8 25795410

[54] Blank CU Enk A. Therapeutic Use of Anti-CTLA-4 Antibodies. Int Immunol (2015) 27(1):3–10. 10.1093/intimm/dxu076 25038057

[55] Robert C Schachter J Long GV Arance A Grob JJ Mortier L. Pembrolizumab *Versus* Ipilimumab in Advanced Melanoma. N Engl J Med (2015) 372(26):2521–32. 10.1056/NEJMoa1503093 25891173

[56] Larkin J Chiarion-Sileni V Gonzalez R Grob JJ Rutkowski P Lao CD. Five-Year Survival With Combined Nivolumab and Ipilimumab in Advanced Melanoma. N Engl J Med (2019) 381(16):1535–46. 10.1056/NEJMoa1910836 31562797

[57] Reck M Schenker M Lee KH Provencio M Nishio M Lesniewski-Kmak K. Nivolumab Plus Ipilimumab *Versus* Chemotherapy as First-Line Treatment in Advanced Non-Small-Cell Lung Cancer With High Tumour Mutational Burden: Patient-Reported Outcomes Results From the Randomised, Open-Label, Phase III CheckMate 227 Trial. Eur J Cancer (2019) 116:137–47. 10.1016/j.ejca.2019.05.008 31195357

[58] Hellmann MD Paz-Ares L Bernabe Caro R Zurawski B Kim SW Carcereny Costa E. Nivolumab Plus Ipilimumab in Advanced Non-Small-Cell Lung Cancer. N Engl J Med (2019) 381(21):2020–31. 10.1056/NEJMoa1910231 31562796

[59] John T Sakai H Ikeda S Cheng Y Kasahara K Sato Y. 1311p First-Line (1L) Nivolumab (NIVO) + Ipilimumab (IPI) + Chemotherapy (Chemo) in Asian Patients (Pts) With Advanced non-Small Cell Lung Cancer (NSCLC) From CheckMate 9la. Ann Oncol (2020) 31:S847–8. 10.1016/j.annonc.2020.08.1625

[60] Reck M Ciuleanu T-E Dols MC Schenker M Zurawski B Menezes J. Nivolumab (NIVO) + Ipilimumab (IPI) + 2 Cycles of Platinum-Doublet Chemotherapy (Chemo) *vs* 4 Cycles Chemo as First-Line (1L) Treatment (Tx) for Stage IV/recurrent Non-Small Cell Lung Cancer (NSCLC): CheckMate 9la. J Clin Oncol (2020) 38(15_suppl):9501–1. 10.1200/JCO.2020.38.15_suppl.9501

[61] Rodriguez-Abreu D Johnson ML Hussein MA Cobo M Patel AJ Secen NM. Primary Analysis of a Randomized, Double-Blind, Phase II Study of the Anti-TIGIT Antibody Tiragolumab (Tira) Plus Atezolizumab (Atezo) *Versus* Placebo Plus Atezo as First-Line (1L) Treatment in Patients With PD-L1-Selected NSCLC (CITYSCAPE). J Clin Oncol (2020) 38(15_suppl):9503–3. 10.1200/JCO.2020.38.15_suppl.9503

[62] Carbone DP Reck M Paz-Ares L Creelan B Horn L Steins M. First-Line Nivolumab in Stage IV or Recurrent Non-Small-Cell Lung Cancer. N Engl J Med (2017) 376(25):2415–26. 10.1056/NEJMoa1613493 PMC6487310 28636851

[63] Wang Z Duan J Cai S Han M Dong H Zhao J. Assessment of Blood Tumor Mutational Burden as a Potential Biomarker for Immunotherapy in Patients With Non-Small Cell Lung Cancer With Use of a Next-Generation Sequencing Cancer Gene Panel. JAMA Oncol (2019) 5(5):696–702. 10.1001/jamaoncol.2018.7098 30816954 PMC6512308

[64] Cascone T William WN Weissferdt A Lin HY Leung CH Carter BW. Neoadjuvant Nivolumab (N) or Nivolumab Plus Ipilimumab (NI) for Resectable Non-Small Cell Lung Cancer (NSCLC): Clinical and Correlative Results From the NEOSTAR Study. J Clin Oncol (2019) 37(15_suppl):8504–4. 10.1200/JCO.2019.37.15_suppl.8504

[65] Toi Y Sugawara S Sugisaka J Ono H Kawashima Y Aiba T. Profiling Preexisting Antibodies in Patients Treated With Anti-PD-1 Therapy for Advanced Non-Small Cell Lung Cancer. JAMA Oncol (2019) 5(3):376–83. 10.1001/jamaoncol.2018.5860 PMC6439838 30589930

[66] Dong ZY Zhong WZ Zhang XC Su J Xie Z Liu SY. Potential Predictive Value of TP53 and KRAS Mutation Status for Response to PD-1 Blockade Immunotherapy in Lung Adenocarcinoma. Clin Cancer Res (2017) 23(12):3012–24. 10.1016/j.jtho.2016.11.504 28039262

